# PARG is dispensable for recovery from transient replicative stress but required to prevent detrimental accumulation of poly(ADP-ribose) upon prolonged replicative stress

**DOI:** 10.1093/nar/gku505

**Published:** 2014-06-07

**Authors:** Giuditta Illuzzi, Elise Fouquerel, Jean-Christophe Amé, Aurélia Noll, Kristina Rehmet, Heinz-Peter Nasheuer, Françoise Dantzer, Valérie Schreiber

**Affiliations:** 1 Biotechnology and Cell Signalling, UMR7242 CNRS, Université de Strasbourg, IREBS, Laboratory of Excellence Medalis, Equipe Labellisée Ligue contre le Cancer, ESBS, 300 Blvd Sébastien Brant, CS 10413, 67412 Illkirch, France; 2 Centre for Chromosome Biology, School of Natural Sciences, National University of Ireland Galway, Galway, Ireland

## Abstract

Poly(ADP-ribosyl)ation is involved in numerous bio-logical processes including DNA repair, transcription and cell death. Cellular levels of poly(ADP-ribose) (PAR) are regulated by PAR polymerases (PARPs) and the degrading enzyme PAR glycohydrolase (PARG), controlling the cell fate decision between life and death in response to DNA damage. Replication stress is a source of DNA damage, leading to transient stalling of replication forks or to their collapse followed by the generation of double-strand breaks (DSB). The involvement of PARP-1 in replicative stress response has been described, whereas the consequences of a deregulated PAR catabolism are not yet well established. Here, we show that PARG-deprived cells showed an enhanced sensitivity to the replication inhibitor hydroxyurea. PARG is dispensable to recover from transient replicative stress but is necessary to avoid massive PAR production upon prolonged replicative stress, conditions leading to fork collapse and DSB. Extensive PAR accumulation impairs replication protein A association with collapsed forks resulting in compromised DSB repair via homologous recombination. Our results highlight the critical role of PARG in tightly controlling PAR levels produced upon genotoxic stress to prevent the detrimental effects of PAR over-accumulation.

## INTRODUCTION

Poly(ADP-ribosyl)ation (PARylation) is a post-translational modification of proteins mediated by Poly(ADP-ribose) polymerases (PARPs). PARylation is involved in numerous biological processes including regulation of transcription and maintenance of genome integrity. The founding member of the PARP family PARP-1 is a key regulator of DNA damage repair, by controlling the recruitment or repellence of DNA repair enzymes as well as chromatin structure modifiers to accelerate repair (1,2). PARylation is a reversible modification, PAR catabolism is mediated mainly by poly(ADP-ribose) glycohydrolase (PARG), encoded by a single gene but present as multiple isoforms localized in different cellular compartments (3,4). In mice, the disruption of all PARG isoforms is embryonic lethal (5). In contrast, in cell-based models, the depletion of all PARG isoforms using either siRNA or shRNA strategies does not necessarily affect cell viability in unstressed conditions. However, upon genotoxic insults, these PARG-deficient cells revealed increased cell death and impaired repair of single- and double-strand breaks (SSB and DSB, respectively) and of oxidized bases (6–8), thereby highlighting the key functions of PARG, like PARP-1, in DNA damage response.

DNA damage response pathways are also activated upon DNA replication stress, leading to stalling of replication forks and activation of S-phase checkpoint. If stalling is transient, the stalled replication fork needs to be stabilized, and replication resumes once the inhibitory signal is removed. Persistent stalling can lead to fork collapse with the dissociation of the replication machinery and the generation of DSB (9). Replication resumes by the opening of new origins and by the repair of DSB through homologous recombination (HR). While a transient short treatment (<6 h) with the ribonucleotide reductase inhibitor hydroxyurea (HU), that deprives the pool of nucleotides, has been shown to trigger transient fork stalling, a longer HU treatment triggers fork collapse and DSB formation (10).

PARP-1^−/−^ mouse embryonic fibroblasts, but also PARP-1-depleted or PARP-inhibited human or mouse cells were shown to be sensitive to HU or triapine, two potent ribonucleotide reductase inhibitors (11–15). PARP-1 was reported to favor replication restart from prolonged stalling of replication fork by recruiting the DNA resection enzyme MRE11 in a PAR-dependent manner (12). However, PARP-1 is not directly involved in the process of DSB repair by HR (11,12,16). In contrast, in conditions of short HU treatment, PARP activity is not required to relocate MRE11 to transiently stalled forks, but, together with BRCA2, protects the forks from extensive MRE11-dependent resection (17). PARP-1 and its activity are also involved in the fork slowing down upon topoisomerase I poisoning with camptothecin (18). At very low concentrations of camptothecin, conditions still sufficient to trigger fork slowing down with the accumulation of regressed forks, PARP-1 activity is critical to protect the regressed forks from a premature RECQL1 helicase-mediated reversion, thus preventing the generation of DSB (19,20).

Although the requirement for PARP-1 and PAR in the response to transient or prolonged replication stress is well established from all the studies described above, it is, however, not known whether a deregulation of PAR catabolism would affect these processes. The role of PARG in response to replicative stress has not been clearly addressed yet. The localization of PARG to replication foci throughout S-phase together with the interaction of PARG with PCNA suggests that PARG could be involved in a replication-related process (21). Murine Parg^−/−^ hypomorphic ES cells (generated by disruption of exon 1) as well as a PARG-depleted human pancreatic cancer cell line showed increased S-phase arrest and increased DSB formation associated with PAR accumulation after treatment with an alkylating agent, suggesting enhanced replication stress (22). Hypomorphe murine Parg^Δ2,3−/−^ cells (generated by disruption of exons 2 and 3) showed persistence of RAD51-foci triggered by a short (6 h) HU treatment (23) but these cells are not completely devoid of nuclear PAR degradation and do not accumulate PAR (24). Additionally, enhanced spontaneous replication fork collapse was reported upon cell treatment with gallotannin (25), an efficient *in vitro* but still questioned *in vivo* PARG inhibitor (26). However, the mechanistic implication of PARG in the recovery from replication stress has not been examined so far.

In this study, we examined the consequences of PARG deficiency in the cell response to transient and prolonged replicative stress triggered by HU. We provide biochemical and cellular evidences that PARG activity is not involved in the recovery from transiently stalled replication forks but is critical to prevent the massive and detrimental accumulation of PAR molecules in conditions of replication fork collapse and generation of DSB.

## MATERIALS AND METHODS

### Cell culture and treatments

Cell lines were maintained in Dulbecco's Modified Eagle's Medium (DMEM 1 g/l glucose) (Invitrogen) supplemented with 10% fetal bovine serum, 1% gentamicin (Invitrogen) and 125 μg/ml hygromycin B under 5% CO2. Stable PARG knockdown (shPARG/PARG^KD^) and control (shCTL/BD650) HeLa cell lines have been described previously (6,27). The cellular clone U2OS-DR-GFP stably transfected with mCherry *-I-Sce*I-GR (28) used for the HR efficiency assay was cultured in DMEM (4.5 g/l glucose) without phenol-red (Invitrogen) supplemented with 10% fetal bovine serum, 100 U/ml penicillin/streptomycin, 0.4 mg/ml G418 (Sigma) and 1 mg/ml puromycin. mCherry positive cells were sorted using FACSAria (Becton Dickinson). Hydroxyurea (HU, Sigma) is diluted in water and added to the cell culture medium at the indicated concentration for the indicated time. Cells are washed twice with PBS at the end of the treatment. PARP inhibitor (KU0058948) (29) was added in complete culture medium to a final concentration of 200 nM, 1 h before HU treatment and maintained throughout the experiment. NU7441 (10 μM; Selleckchem) or mirin (20 μM; Sigma) were added in complete medium 2 h before HU treatment and maintained throughout the experiment.

### Colony-forming assay

Four thousand shPARG or shCTL cells were seeded in triplicate on 100-mm dishes 5 h prior to treatment with the indicated dose of HU in complete medium for 24 h. Cells were washed twice with PBS and incubated for 11 days in complete medium. Colonies were fixed in 3.7% formaldehyde, stained with 0.1% crystal violet and enumerated using ImageJ (NIH, Bethesda, MD, USA).

### Flow cytometry

Five hundred thousand cells were seeded in 100-mm culture dishes and treated the following day with the indicated dose of HU for either 4 or 24 h. For cell cycle distribution analyses (FACS), cells were trypsinized, fixed in ethanol 70% for 24 h and rehydrated in PGE (PBS, 1 g/l glucose, 1 mM EDTA). Nuclear DNA was stained with propidium iodide (Sigma; 4 μg/ml) in the presence of RNase A (Sigma; 10 μg/ml) in PGE for 30 min. Stained cells were analysed on an FACScalibur (Becton Dickinson) using CellQuest software (Becton Dickinson). Ten thousand cells gated as single cells were analysed. For analyses of PAR production, cells were collected, fixed and rehydrated as above. Cells were incubated 20 min in 2 ml PTB buffer (PBS, 0.5% Tween-20, 0.5% BSA) then 2 h at RT with the mouse monoclonal anti-PAR antibody (10H, 1:200 in PTB). After two washes with PTB, cells were incubated 1 h at RT with Alexa 488 conjugated goat anti-mouse IgG3k secondary antibodies (Invitrogen) diluted 1:500 in PTB. After two washes in PTB, nuclear DNA was stained with propidium iodide and cells analysed by flow cytometry as described above.

### Immunofluorescence

Cells grown on glass coverslips were treated or not, as indicated in the figure legends, then processed as follows: washed in PBS and fixed 20 min in ice-cold methanol:acetone (v:v). For RPA2 (replication protein A (RPA) subunit 2), P-S4S8-RPA2, RAD51 and MRE11 immunodetection, a pre-extraction step was carried out by incubating the coverslips in CSK extraction buffer (50 mM HEPES, pH 7.5, 150 mM NaCl, 1 mM EDTA) containing 0.3% Triton X-100 on ice two times for 5 min before fixation. Permeabilization was done in PBS, 0.1% Tween. Cells were incubated overnight at 4°C with PBS, 0.1% Tween, containing 1 mg/ml BSA and a primary antibody: mouse monoclonal anti-PAR 10H (IgG_3_κ, 1:1000), anti-γH2AX (Ser139) (IgG_1_, 1:2000, Upstate), anti-BrdU (IgG_1_, 1:7, Becton Dickinson), rabbit monoclonal anti-P-Chk1 (S345) (cl133D3, IgG, 1:400, Cell Signaling), rabbit polyclonal IgG anti-MRE11 (NB100–142, 1:400, Novus Biologicals), anti-RAD51 (H-92) (1:1000, Santa Cruz Biotechnology), anti-RPA2 (ab2626) (1:2000, Abcam), anti-P-RPA2 (S4S8) (1:4000, Bethyl Laboratories). After washing, cells were incubated for 2 h with the appropriate secondary antibodies: an Alexa Fluor 488 or 568 goat anti-mouse IgG or IgG1 or IgG3, or an Alexa Fluor 488 or 568 goat anti-rabbit IgG (1:2000, Molecular Probes, Invitrogen). After three washes with PBS, 0.1% Tween, DNA was counterstained with Dapi (25 ng/ml in PBS) and slides were mounted in Mowiol (Sigma) containing the anti-fading agent Dabco (Sigma). Immunofluorescence microscopy was performed using a Leica DMRA2 microscope (Leica Microsystems) equipped with an ORCA-ER chilled CCD camera (Hamamatsu) and the capture software Openlab (Improvision). Merging of images was done using ImageJ.

### Single-stranded DNA labeling by BrdU immunodetection

The experiment was performed essentially as described (30). Briefly, cells on glass coverslips were grown in the culture medium containing 10 μM BrdU (Sigma) for 40 h, corresponding to two cell cycles. BrdU were removed with PBS washes and fresh medium with or without 2 mM HU was added, for 24 h. For the release points, cells were washed with PBS and fresh medium was added for the indicated release times. To finally detect single-stranded DNA (ssDNA), the coverslips were processed for immunofluorescence detection using anti-BrdU and anti-PAR antibodies, as described above. To verify BrdU incorporation into the entire nuclear DNA, a denaturation step prior to permeabilization was introduced, comprising a 15-min incubation with 3 N HCl on ice and washes with 0.1 M Na_2_B_4_O_7_ −10H_2_O, pH 8.5.

### Western blot

Total cell lysates were prepared by adding Laemmli buffer (4% SDS, 20% glycerol, 120 mM Tris-HCl pH 6.8) directly on the culture dishes, sonication of the collected samples and estimation of the relative protein content by analysing an aliquot on a 10% SDS-PAGE. The gel was stained with Coomassie Blue followed by analysis of staining intensity of some common strong bands using a LICOR Odyssey. After normalization, equal amounts of proteins were analysed by western blotting using the appropriate antibodies: mouse monoclonal anti-α-tubulin (Sigma), anti-γH2AX (Ser139) (Upstate), anti-Chk1 (G-4) (Santa Cruz Biotechnology), anti-RPA2 (ab2175, Abcam), anti-PARP-1 (EGT69, in-house production), rabbit polyclonal anti-PAR (Trevigen), anti-P-Chk1 (S345) (Cell Signaling), anti-RPA2 (ab2626, Abcam), anti-P-S4S8-RPA2 and anti-P-S33-RPA2 (Bethyl Laboratories), anti-MRE11 (NB100–142) (Novus Biologicals), anti-RAD51 (H-92) (Santa Cruz Biotechnology), anti-GAPDH (Sigma) and the rabbit monoclonal anti-histone H4 (Millipore). After washings, membranes were incubated for 1 h with the appropriate secondary antibodies: an Alexa Fluor 680 goat anti-rabbit or goat anti-mouse Alexa Fluor 680 (1:20 000, Invitrogen) or a Donkey anti-mouse IRDye800 (1:10 000, Li-Cor, Bioscience) and revealed on Odyssey Infrared Imaging System (Li-Cor, Bioscience). Quantification was performed with the analysis software provided.

### Biochemical fractionation

After different treatments, cells were washed with PBS and harvested. Pellets of 5 × 10^6^ cells were fractionated as previously reported (31) with minor modifications. Briefly, cells were first resuspended for 5 min on ice in 200 μl of fractionation buffer (50 mM HEPES, pH 7.5, 150 mM NaCl, 1 mM EDTA) containing 0.05% Nonidet P-40 (NP-40) and supplemented with 1 mM Pefabloc (Roche), 1 mM Na_3_VO_4_ and 50 mM NAF. Following centrifugation at 1000 × *g* for 5 min, the supernatant was collected (fraction I), and pellets were incubated in 200 μl of the same buffer supplemented with 100 mg/ml RNase A (Sigma) for 10 min at 20°C. The supernatant was collected as before (fraction II), and the nuclear pellets were further extracted for 40 min on ice with 200 μl of fractionation buffer containing 0.5% Nonidet P-40. The extracts were clarified by centrifugation at 16 000 × *g* for 15 min (fraction III). The pellets were resuspended in 200 μl extraction buffer supplemented with 1% Triton X-100 and 450 mM NaCl and sonicated (Bioruptor nextgen, Diagenode). Same aliquots of the fractions I–IV, derived from equivalent cell numbers for each culture conditions, were added to loading buffer, boiled and loaded on SDS–PAGE and then analysed by western blot as described above. Odyssey Infrared Imaging System was used for detection. GAPDH (glyceraldehyde 3-phosphate dehydrogenase) was used as a marker of the cytosolic and early extracted fractions, whereas histone H4 was used as a marker for the last fraction IV, enriched in chromatin (Supplementary Figure S1).

### Immunoprecipitation

For immunoprecipitation experiments, shCTRL and shPARG cells were treated with 2 mM HU for 24 h. Cells were collected and resuspended in EBC lysis buffer (50 mM Tris-HCl pH 8, 120 mM NaCl, 0.1% NP-40, 1 mM EDTA, 50 mM NaF, 1 mM Na_3_VO_4_ and 1 mM Pefabloc), incubation in ice for 20 min followed by centrifugation at 14 000 × *g* at 4°C for 5 min. Immunoprecipitation was performed on 1 mg of whole cell extracts. After a preclearing with protein A Sepharose (GE Healthcare) for 1 h at 4°C, the cleared suspensions were incubated with either purified polyclonal rabbit antibody anti-PAR (1:50, Trevigen) or rabbit anti-mouse antibody as control; for RPA2 immunoprecipitation 4 μg of the mouse monoclonal anti-RPA2 antibody (ab2175, Abcam) or anti-HA (Santa Cruz) as control was used, and left in rotation overnight at 4°C, followed by 3-h incubation at 4°C with protein A Sepharose. Beads were recovered by centrifugation at 4000 rpm at 4°C for 5 min and washed three times with the EBC buffer supplemented with inhibitors. Final pellets were resuspended in Laemmli buffer and boiled 5 min. Samples were finally loaded onto 10% SDS-PAGE and analysed by western blot as described previously.

### *In vitro* PARylation and PAR binding assays

Purified PARP-1 (500 ng) was incubated with 2.5 μg of RPA or 3 μg of GST at 25°C for 30 min in 35 μl of 50 mM Tris-HCl pH7.5 containing 0.5 μCi [^32^P]-NAD^+^ and 500 ng of DNAse I treated calf thymus DNA. Reaction products were separated on 8–20% gradient SDS-PAGE, proteins were stained with Coomassie Blue and exposed to PhosphorImager screens and analysed with a Typhoon™ FLA 9500 biomolecular imager (GE Healthcare). For PAR binding (PAR-blot), RPA (2.5 μg), GST (2 μg) and core histones (1 μg each) were separated on 8–20% gradient SDS-PAGE and either stained by Coomassie Blue or transferred onto nitrocellulose membrane. The membrane was incubated 2 h at 25°C in PBS with non-radioactive PAR prepared as described (21). After extensive washing with PBS supplemented with 500 mM NaCl, the membrane was successively probed with 10H anti-PAR antibody (1:100) and Alexa Fluor 680 goat anti mouse antibody (1:20 000) and revealed using an Odyssey Infrared Imaging System.

### Electrophoretic mobility shift assay

Assays were performed in 20 μl reaction in PBS at room temperature. For DNA and PAR pre-incubation experiments, 1 nM of an HPLC purified 50-mer 5′ labeled Cy5.5 OligodT (Cy5.5-oligodT) DNA substrate (Sigma) was mixed with increasing amount of competing PAR (32 ng/μl) as indicated in figure legend, before addition of 1.7 nM of RPA and incubation 30 min at 20°C. For DNA and RPA pre-incubation experiments, RPA (7 nM) was pre-incubated with 2.5 nM of the Cy5.5-oligodT probe for 15 min before the addition of increasing amount of PAR (from 4 to 192 ng, as indicated in the figure legend) and incubation for 30 min at 23°C. Five microliters of loading buffer (40% glycerol, 0.01% Bromophenol blue in TBE 0.5×) were added prior to electrophoresis (5 V/cm) on 1% agarose gel in TBE 0.5× at 4°C for approximately 2 h. The fluorescent DNA was revealed by scanning the gel at 700 nm using an Odyssey Infrared Imaging System. Bands intensities were assessed with ImageJ.

### Homologous recombination

HR was performed as previously described (28). Briefly, U2OS cells containing the HR reporter DR-GFP and the inducible mCherry*-I-Sce*I-GR (U2OS-DR-GFP- mCherry*-I-Sce*I-GR) were transfected with 25 nM of the indicated siRNA (Dharmacon) with jetPRIME (Polyplus-transfection, France) following the manufacturer's instructions. Twenty-four hours after, the same transfection was repeated and after further 48 h, cells were treated with 100 ng/ml of triamcinolone acetonide (TA, Sigma) to induce nuclear translocation of the mCherry*-I-Sce*I-GR. Cells were harvested 2 days after and subjected to flow cytrometry analysis to examine recombination frequency by evaluation of GFP-positive cells out of the mCherry-positive cells. FACS analyses were done using the FACSCalibur and Cell Quest software (Becton Dickinson). Efficiency of the RNA depletion was verified by western blot or RT-qPCR (reverse transcription-quantitative polymerase chain reaction).

### Statistical analysis

All experiments were performed at the minimum in triplicate, unless otherwise stated. Data are expressed as means ± SD and were obtained by one-way analysis of variance followed by the Student's *t*-test. *P* values are indicated in the legend of each figure.

## RESULTS

### PARG-deficient cells display impaired S-phase progression upon long but not short HU exposure

**Figure 1.**
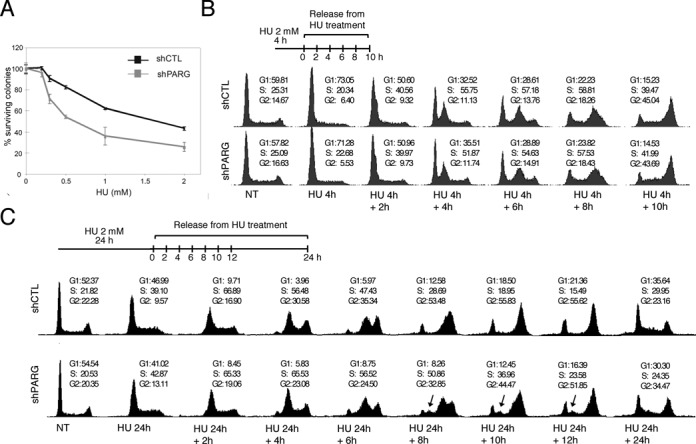
PARG-depleted cells are affected in S-phase progression after long but not short HU exposures. (**A**) Increased sensitivity of shPARG cells to HU. Cell survival analysis of shCTL (black) and shPARG (gray) cell lines after 24-h treatment with increasing concentrations of HU. Experiment shown is a representative out of four with each condition done in triplicate. Results are means ± SEM. (**B**) PARG is dispensable for S-phase recovery and progression after short HU treatments. shCTL and shPARG cells were treated with 2 mM HU for 4 h, released into fresh medium (0 h, labeled as HU 4 h) for the indicated time points and their DNA content was analysed by flow cytometry. The percentage of cells in G1, S, G2/M (noted G2) is indicated. (**C**) PARG depletion impacts S-phase restart and progression after a prolonged HU treatment. shCTL and shPARG cells were treated with 2 mM HU for 24 h, released into fresh medium (0 h, labeled as HU 24 h) and analysed as in panel B. Untreated logarithmically growing cells are presented as controls (NT).

The impact of PARG deficiency on the replicative stress response was evaluated in HeLa cells constitutively depleted of all PARG isoforms (shPARG) and their respective control (shCTL) described previously in Amé *et al.* (6). The shPARG cell line displayed a decreased cell survival compared to shCTL after treatment with HU at concentrations ranging from 0.25 to 2 mM for 24 h (Figure 1A), indicating that PARG deficiency sensitizes human cells to replicative stress.

To investigate whether the higher sensitivity of the shPARG cells to HU was due to defects in the recovery from either stalled or collapsed forks, we compared the cell cycle distribution of shPARG and shCTL cell lines after HU treatments of different lengths. In the absence of treatment, the cell cycle profile of shPARG and shCTL cells was similar, indicating that PARG is dispensable for DNA replication in unstressed conditions. Short HU treatment (less than 6 h) transiently stalls replication forks whereas long HU treatment (12–24 h) triggers replication fork collapse and generation of DSB (9,10). A similar cell cycle profile was observed for shPARG and shCTL at any time point after the release from a short treatment with 2 mM HU for 4 h (Figure 1B). In contrast, shPARG cells treated with HU for a long time (2 mM for 24 h) showed a significant defect in S-phase recovery (Figure 1C): S-phase was globally slowed down (a 2-h delay is seen at 8 and 10 h post-release) and a G2/M arrest was observed 24 h after the release. In addition, some cells could not resume cell cycle progression after the prolonged HU treatment (see arrows). These results suggest that PARG is dispensable for the restart of stalled replication forks but required for efficient recovery from replicative stress conditions known to trigger replication fork collapse.

### Increased PAR formation and γH2AX signal in HU-treated PARG-deficient cells

**Figure 2.**
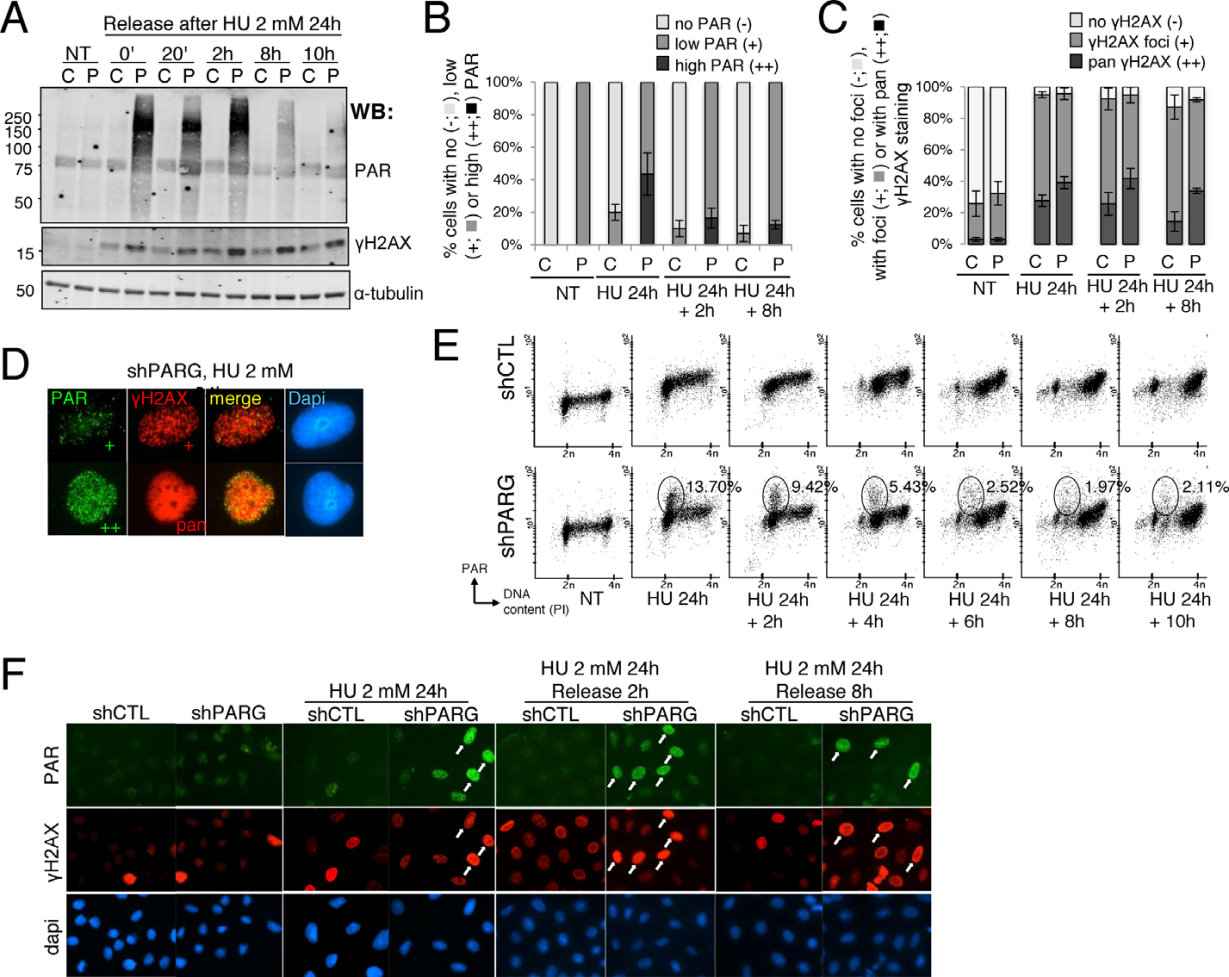
Increased PAR synthesis and H2AX phosphorylation in HU-treated shPARG cells. (**A**) Massive and persistent accumulation of PAR and enhanced phosphorylation of γH2AX in shPARG cells treated with 2 mM HU for 24 h and release into fresh medium for the indicated time points. Equivalent amount of total cell extracts was analysed by western blotting using the indicated antibodies. C: shCTL; P: shPARG; NT: untreated. The band above 75 kDa detected with the anti-PAR antibody in all samples is non-specific. (**B**) Evaluation by immunofluorescence of PAR cellular levels in shCTL and shPARG cells either untreated (NT) or treated with 2 mM HU for 24 h (HU 24 h) and further released for 2 or 8 h as indicated in the figure. The histogram depicts the percentage of cells displaying no PAR staining (−, light gray bar), low levels of PAR (+, gray bar, see D) or very high levels of PAR (++, black bar, see D). An average of 500 cells per condition were scored in randomly selected fields. Mean values of seven independent experiments ± SD are shown. (**C**) Evaluation by immunofluorescence of γH2AX cellular levels in shCTL and shPARG cells either untreated (NT) or treated with 2 mM HU for 24 h (HU 24 h) and further released for 2 or 8 h as indicated in the figure. The histogram depicts the percentage of cells displaying no γH2AX staining (−, light gray bar), γH2AX foci (+, gray bar, see D) or pan γH2AX staining (++, black bar, see D). An average of 500 cells per condition were scored in randomly selected fields. Mean values of three independent experiments ± SD are shown. (**D**) Representative immunofluorescence images of the PAR and γH2AX cellular level classifications, taken from shPARG cells treated with 2 mM HU for 24 h: low PAR (+, green), high PAR (++, green), γH2AX foci (+, red) and pan γH2AX staining (++, red). The anti-PAR and anti-γH2AX signals are merged, showing no significant co-localization. The nuclei are counterstained with Dapi. (**E**) PAR production in HU-treated shPARG visualized by flow cytometry. shCTL and shPARG cells treated with 2 mM HU for 24 h and released in fresh medium for the indicated times were subjected to immunolabeling of PAR using mouse monoclonal antibody, DNA staining with propidium iodide, and analysed by flow cytometry. The percentage of high PAR producing cells blocked in early S-phase after the release from HU treatment is calculated from the ellipse presented in the FACS diagrams. (**F)** The enhanced PAR and γH2AX produced in HU-treated shPARG cells is confirmed by immunofluorescence. Immunodetection of PAR and γH2AX with appropriated antibodies in fixed shCTL and shPARG cells treated or untreated with 2 mM HU for 24 h and released for 2 or 8 h as indicated in the figure. Nuclear DNA is counterstained with Dapi. White arrows point to high PAR cells positive for γH2AX staining.

To investigate whether the S-phase delay upon long HU treatment was associated with an accumulation of prolonged stalled forks and DSB, we analysed PAR formation and γH2AX accumulation. Although PAR could not be detected in shCTL cells by western blot after treatment with 2 mM HU for 24 h, PARG-deficient cells showed a strong accumulation of PAR that persisted for at least 2 h after the release (Figure 2A). At 8 and 10 h after the release, PAR levels were reduced in shPARG cells but still significantly higher than in shCTL cells. This dissipation of PAR over time could be due to some residual PARG enzymes or to the possible activity of the other PAR-degrading enzyme ARH3 (32). By immunofluorescence, HU-treated shCTL cells mainly showed low levels of PAR production (20 ± 5%, Figure 2B and see high magnification of cells in Figures 2D and 6A) while HU-treated shPARG cells displayed higher levels of PAR, with 43 ± 13% of cells characterized by a very strong PAR accumulation, a typical sub-population not present in the HU-treated shCTL cells. After release from the HU treatment, PAR levels gradually decreased in both cell lines, with some shPARG cells still maintaining high levels of PAR (16 ± 6% and 12 ± 3%, 2 and 8 h after the release from the HU treatment, respectively, Figure 2B). In the subsequent experiments of this study, we categorized the shPARG cells according to their PAR levels, either displaying no or low PAR (noted as PAR−/+), or high PAR (noted as PAR++). For shCTL cells, we used a unique PAR−/+ (no or low PAR) category. FACS analyses confirmed that a significant proportion of the HU-treated shPARG cells blocked in early S-phase produced high levels of PAR up to 6 h after the release (Figure 2E). Immunodetection of γH2AX by western blot (Figure 2A) and by immunofluorescence (Figure 2C and D) showed that both cell lines responded to HU by the phosphorylation of H2AX that was maintained several hours after the release but shPARG cells showed higher levels of γH2AX compared to shCTL cells. The shPARG cells displaying high PAR levels also showed a strong γH2AX staining throughout the release from HU treatment (Figure 2F). Of note, even in cells displaying discrete γH2AX foci, no significant co-localization with PAR could be noticed (Figure 2D). Collectively, these results suggest that in shPARG cells, prolonged replicative stress leads to higher levels of DSB arising from replication fork collapse accompanied by the accumulation of γH2AX and PAR.

**Figure 3.**
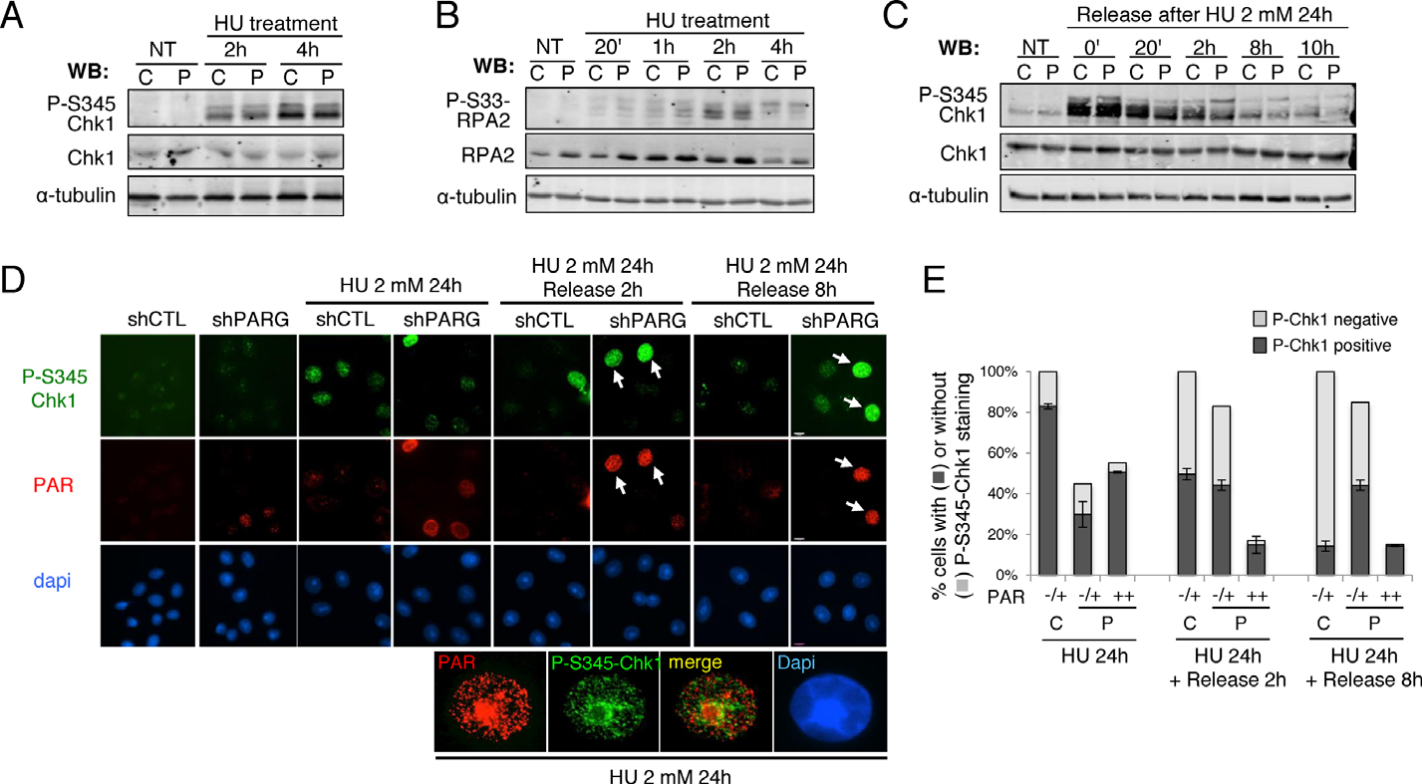
Normal checkpoint activation in HU-treated shPARG cells, but persistent checkpoint activation in high PAR cells after prolonged HU exposure. (**A**) Efficient phosphorylation of Chk1 on S345 in shPARG cells treated with 2 mM HU for 1 or 2 h. Equivalent amount of total cell extracts were analysed by western blotting using the indicated antibodies. C: shCTL; P: shPARG; NT: untreated. (**B**) Normal phosphorylation of RPA2 at S33 in shPARG cells treated with 2 mM HU for the indicated time points, examined by western blotting as described in A using the indicated antibodies. (**C**) Phosphorylation of Chk1 on S345 in shPARG and shCTL cells treated with 2 mM HU for 24 h and released into fresh medium for the indicated time points, and examined by western blotting as described in A using the indicated antibodies. (**D and E**) Persistence of Chk1 phosphorylation on S345 in shPARG with high PAR levels. (**D**) Immunofluorescence of P-S345 Chk1 and PAR with appropriated antibodies in fixed cells that were treated with 2 mM HU for 24 h and released for 2 or 8 h, or left untreated. Nuclear DNA is counterstained with Dapi. White arrows point to high PAR cells positive for P-S345 Chk1 staining. Higher magnifications of the PAR and P-S345-Chk1 immunofluorescence detections in an shPARG cell treated with 2 mM HU for 24 h are shown, with the merged signals showing no significant co-localization. (**E**) Histogram showing the percentage of P-S345-Chk1 stained (black bar) or not (light gray bar) in shCTL or shPARG cells relative to their PAR cellular levels and categorized in either no and low PAR (−/+) or high PAR (++) levels. More than 500 nuclei in each condition were scored, bars represent the mean values measured with ImageJ software from three independent experiments ± SD.

**Figure 4.**
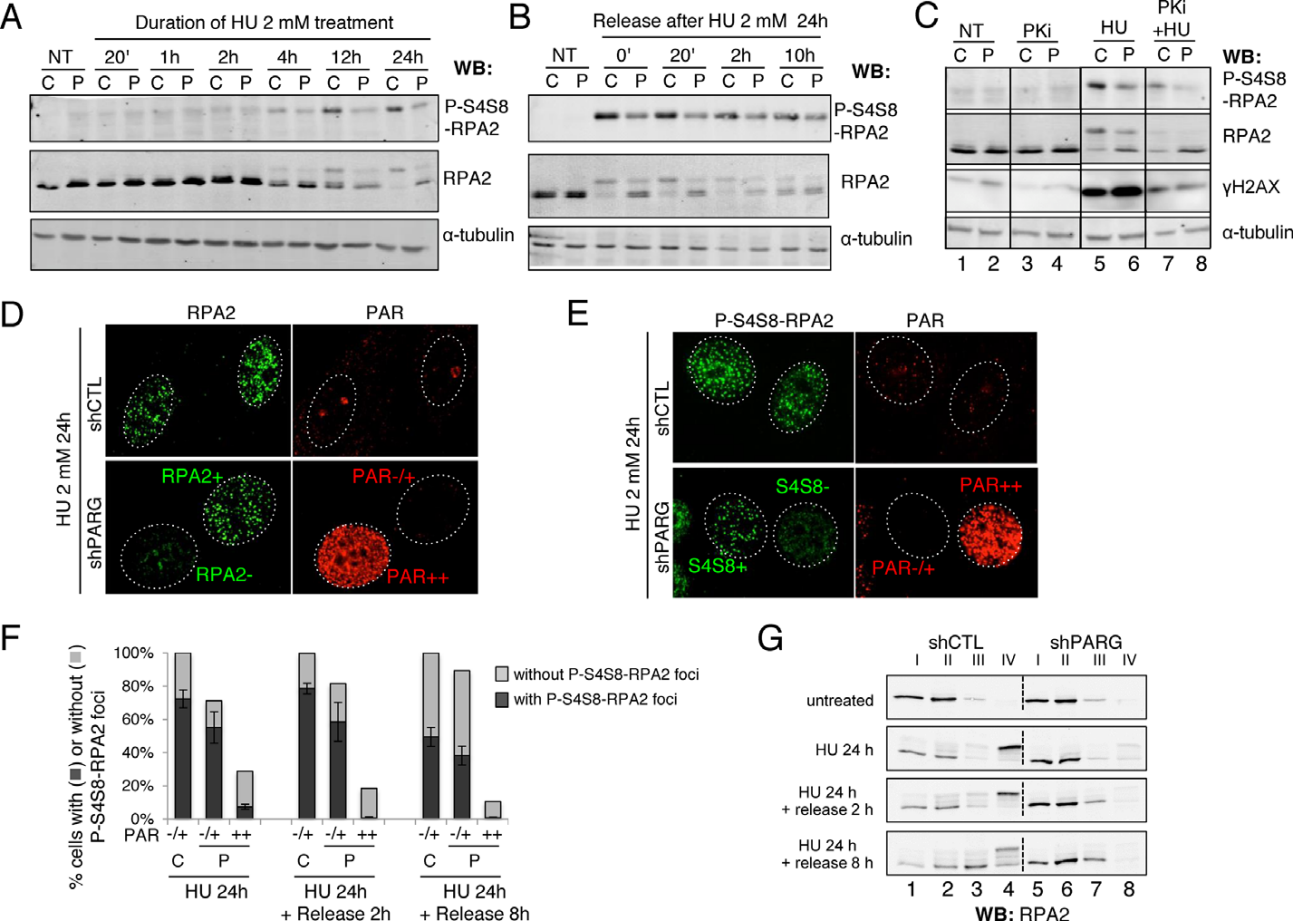
Decreased RPA2 hyperphosphorylation and chromatin loading in HU-treated shPARG cells. (**A**) Decreased phosphorylation of RPA2 on S4S8 in shPARG (P) cells compared to shCTL (C) cells after treatment with 2 mM HU for the indicated time points. Equivalent amounts of total cell extracts were analysed by western blotting using the indicated antibodies. NT: untreated. (**B**) Decreased phosphorylation of RPA2 on S4S8 in shPARG (P) cells compared to shCTL (C) cells after incubation with 2 mM HU for 24 h (0′) and release for the indicated time (20′ to 10 h). Total cell extracts were analysed by western blotting as described in A. (**C**) DNA-PK, which is mainly responsible for the RPA2 phosphorylation at S4S8, is functional in shPARG cells. shCTL (C) and shPARG (P) cells were incubated with 2 mM HU for 24 h in the presence or absence of 10 μM of the specific DNA-PK inhibitor NU7441 (PKi). Equivalent amount of total cell extracts were analysed by western blotting using the indicated antibodies described as in A. (**D**) HU-induced RPA2 foci formation is impaired in PARG-depleted cells with high PAR. shCTL and shPARG cells were treated with 2 mM HU for 24 h and processed for immunofluorescence using anti-RPA2 and anti-PAR antibodies after a pre-extraction step, as described in *“Materials and Methods”* section. (**E**) HU-induced P-S4S8-RPA2 foci formation is impaired in PARG-depleted cells with high PAR. shCTL and shPARG cells were treated with 2 mM HU for 24 h and processed for immunofluorescence using anti-P-S4S8-RPA2 and anti-PAR antibodies as presented in D. (**F**) Histogram depicting the percentage of cells scored positive (black bar) or negative (light gray bar) for P-S4S8-RPA2 foci relative to their PAR cellular level and categorized in either no and low PAR (−/+) or high PAR (++) levels. For each point, bars represent the mean values of >200 nuclei measured with ImageJ software from three independent experiments ± SD. **G.** RPA2 loading onto chromatin is strongly impaired in shPARG cells, which were treated with 2 mM HU for 24 h and further released into fresh medium for 2 and 8 h, or left untreated. Same number of cells was collected and fractionated as described in the “*Material and Methods*” section leading to fractions I to IV. Equivalent cell numbers of each fraction were analysed by western blotting using an anti-RPA2 antibody.

**Figure 5.**
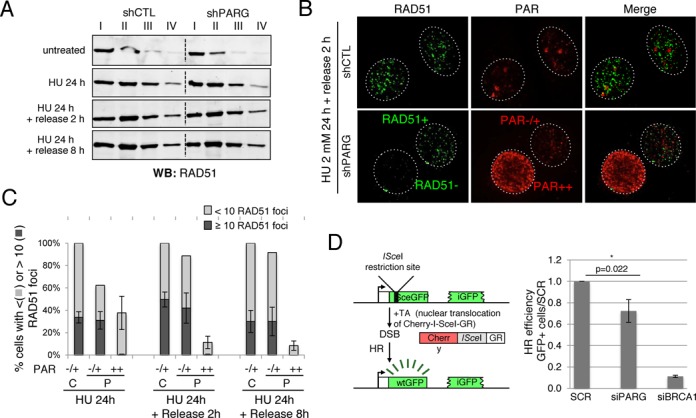
Repair of HU-induced DSB by homologous recombination is affected in high PAR shPARG cells. (**A**) RAD51 loading onto chromatin is globally not affected in shPARG cells treated with 2 mM HU for 24 h and further released into fresh medium for 2 or 8 h. Same number of untreated or HU-treated shCTL and shPARG cells was collected, fractionated as described in the *“Materials and Methods”* section leading to fractions I to IV. Equivalent cell numbers of each fraction were analysed by western blotting using an anti-RAD51 antibody. (**B and C**) Decreased HU-induced RAD51 foci formation in PARG-depleted cells displaying high PAR. shCTL and shPARG cells were incubated with 2 mM HU for 24 h, released into fresh medium for 2 or 8 h and processed for immunofluorescence using anti-RAD51 and anti-PAR antibodies after a pre-extraction step described in “*Material and Methods*” section. (**B**) Representative immunofluorescence image at 2 h after release from HU treatment; cells with high PAR levels (PAR++) display less than 10 RAD51 foci (RAD51−). (**C**) Histogram depicting the percentage of cells with more than 10 RAD51 foci per cell (black bar) or less than 10 RAD51 foci (light gray bar) relative to their PAR cellular level and categorized in either no and low PAR (−/+) or high PAR (++). For each point, bars represent the mean values of >200 nuclei scored for each condition from three independent experiments ± SD. (**D**) HR efficiency is decreased after PARG depletion. The frequency of HR-mediated repair events was analysed by flow cytometry in U2OS-DR-GFP-mCherry-*I-Sce*I-GR cells after transfection with the indicated siRNA and induction of DSB formation by the translocation of mCherry-*I-Sc*eI-GR from cytoplasm to nucleus upon incubation with TA for 48 h. The values correspond to the percentage of GFP-positive cells relative to the control set as 1.0 (cells transfected with scrambled siRNA, SCR) and represent the mean ± SD of five independent experiments: The asterisk * indicates a value of *P* = 0.022 versus SCR.

**Figure 6.**
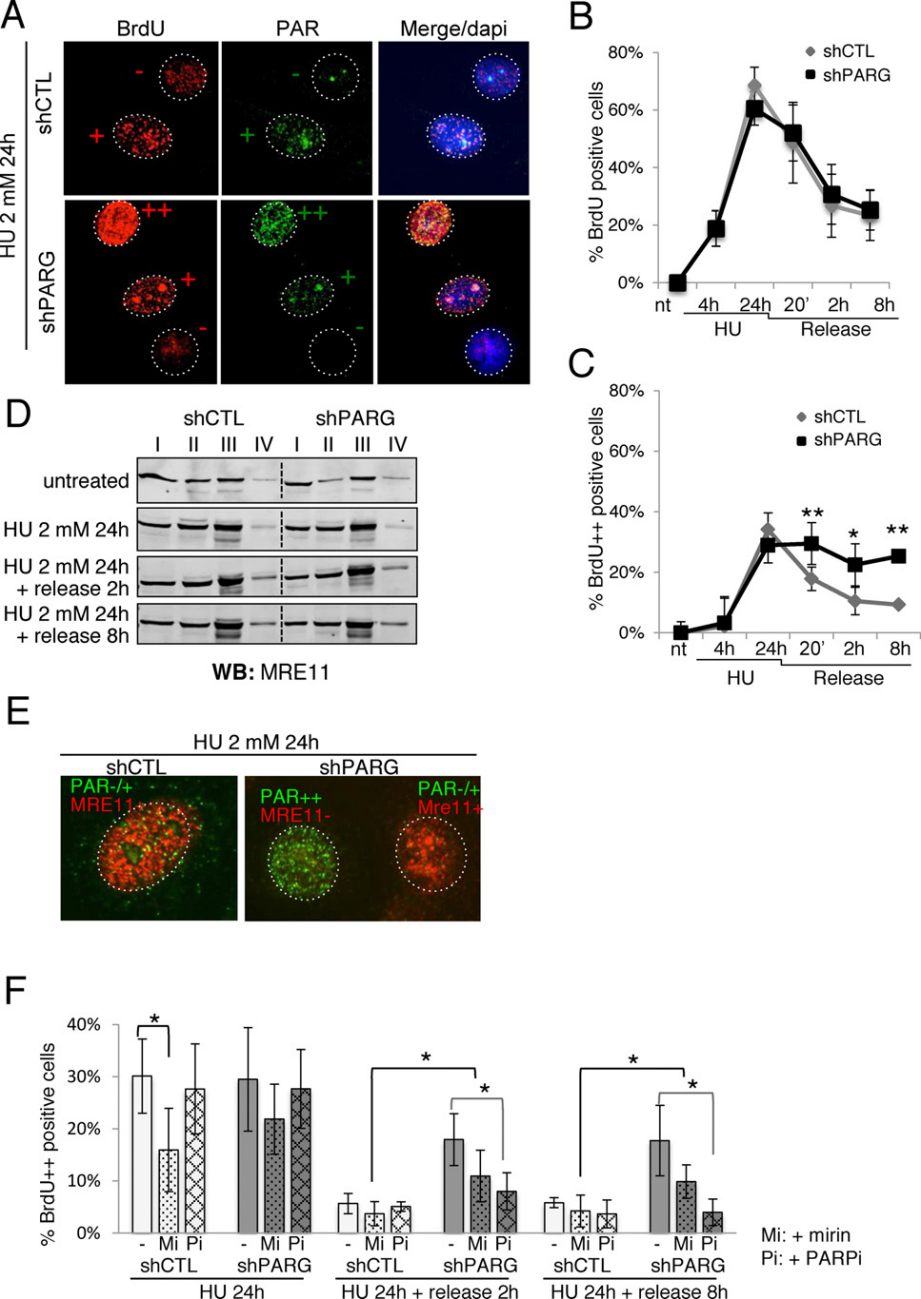
ssDNA persists in shPARG cells with high PAR levels after prolonged exposure to HU. (**A-C**) BrdU (10 μM) was incorporated for two complete cell cycles before the HU treatment (2 mM for 24 h, followed by a release for 20 min, 2 or 8 h in fresh medium) and detected by immunofluorescence using an anti-BrdU antibody, concomitantly with the immunodetection of PAR. (**A**) The shPARG cells with strong BrdU staining (++, red), persisting after the release from prolonged HU treatment, are those displaying high PAR levels (++, green), whereas cells with limited (+, red) or no (−, red) BrdU staining have low (+, green) or no (−, green) PAR, respectively. DNA is counterstained with Dapi. (**B**) The graph illustrates the proportion of BrdU-positive cells detected without DNA denaturation, expressed as percentage of the total cells analysed. More than 500 cells per condition were scored; mean values of three independent experiments ± SD are shown. *P: < 0.05; **: *P* < 0.01. (**C**) The graph depicts the proportion of shCTL and shPARG cells showing a strong BrdU signal without DNA denaturation (noted ++ in A), and scored as in B. (**D**) MRE11 mobilization onto chromatin is globally not affected in shPARG cells incubated with 2 mM HU for 24 h and further released or not into fresh medium for 2 or 8 h. Same number of untreated or HU-treated shCTL and shPARG cells was collected, fractionated as described in the “*Materials and Methods*” section leading to fractions I to IV. Equivalent cell numbers of each fraction were analysed by western blotting using an anti-MRE11 antibody. (**E**) MRE11 foci formation is observed in HU-treated shPARG cells with low but not high PAR levels. shCTL and shPARG cells were treated with 2 mM HU for 24 h and processed for immunofluorescence using anti-MRE11 and anti-PAR antibodies after a pre-extraction step, as described in *“Materials and Methods”* section. Representative cells with low PAR (PAR−/+), high PAR (PAR++), MRE11 foci (MRE11+) and no MRE11 foci (MRE11-) are shown. (**F**) The graph depicts the proportion of cells showing a strong BrdU signal without DNA denaturation (BrdU++), in shCTL and shPARG cells treated with HU 2 mM for 24 h and released for 2 or 8 h in fresh medium. When indicated, the HU treatment and release were performed in the presence of the MRE11 inhibitor mirin (Mi) or the PARP inhibitor KU0058948 (Pi, PARPi). Results are expressed as percentage of the total cells analysed. More than 500 cells per condition were scored; mean values of three independent experiments ± SD are shown. *P: < 0.05.

### Persistence of S-phase checkpoint activation in high PAR HU-treated shPARG cells

The difference between shPARG and shCTL in S-phase recovery from prolonged replicative stress prompted us to examine the activation of the replicative stress checkpoint in response to HU treatment. Activation of this checkpoint relies on the ATR kinase that phosphorylates Chk1 on Serine 345 and the ssDNA-binding protein RPA2 on S33 (33–35). By western blot, we observed similar phosphorylation of Chk1 on S345 (Figure 3A) and of RPA2 on S33 (Figure 3B) in shCTL and shPARG cells in conditions of transient replication stress (HU treatment for 20 min to 4 h), consistent with PARG being dispensable for the activation of checkpoint and recovery from transient replication stress (Figure 1B). After 4 h of HU, hyperphosphorylation of RPA2 was detected with anti-RPA2, anti-P-S33 and anti-P-S4S8 antibodies (Figures 3B and 4A), indicating that fork collapsing started to take place, in agreement with Sirbu *et al.* (36). When the replication stress persisted upon long time (HU treatment 2 mM for 24 h), phosphorylation of Chk1 on S345 was detected by western blot in both HU-treated shCTL and shPARG cells (Figure 3C). A slower migrating band detected with the anti-S345-P-Chk1 antibody remained at higher levels after release in the shPARG cells, suggesting that the checkpoint activation could persist in at least a fraction of the damaged shPARG cells. A detailed analysis by immunofluorescence revealed that whereas the proportion of cells displaying S345-P-Chk1 signal progressively decreased with time after the release from HU-treatment in shCTL cells, it remained higher in the shPARG cells producing limited amounts of PAR (PAR−/+). Only 14 ± 3% of shCTL cells were still positive for S345-P-Chk1 phosphorylation at 8 h, compared to 44 ± 2% of the shPARG cells with low PAR levels (Figure 3D and E), supporting the delay in S-phase progression of shPARG cells released from prolonged HU treatment (Figure 1C). A clear correlation between persistent S345-Chk1 phosphorylation and high PAR level was noticed (Figure 3D, arrows), since the high PAR shPARG cells (PAR++) were almost all P-S345-Chk1 positive (Figure 3D, arrows, and E). Of note, no significant co-localization between PAR and P-S345-Chk1 was noticed (Figure 3D, see high magnification panels). These results support the hypothesis that the cells accumulating high amount of PAR do not resume replication due to persistent checkpoint activation.

### Decreased hyperphosphorylation and chromatin loading of RPA after prolonged HU treatment in shPARG cells

The DSB generated upon fork collapse after long-lasting stall are essentially repaired by the HR pathway, using homologous DNA sequences as a template for re-synthesis of the sequence containing the break (35). First, MRE11 forming a functional complex with RAD50 and NBS1 (the MRN complex) initiates DNA resection starting from the DSB. Resection is further extended on both directions by MRE11, CtIP and Exo1 to generate 5′ overhang ssDNA rapidly coated by the trimeric protein RPA to protect it. RPA2 becomes hyperphosphorylated on S4 and S8, mainly by DNA-PK (33,34,37,38). Therefore, RPA foci formation and hyperphosphorylation are commonly used as readouts of efficient resection (39,40). To evaluate the impact of PARG deficiency in HR, we monitored the phosphorylation of RPA2 on S4S8 in shPARG and shCTL cells following treatment with 2 mM HU for the indicated times (Figure 4A). Whereas S4S8 phosphorylation was strongly induced after 12 and 24 h of HU treatment in shCTL, it was dramatically impaired in shPARG cells (Figure 4A, upper panel). This was correlated with the strong decrease of the slow migrating band detected with anti-RPA2 antibody and representing the hyperphosphorylated form of RPA2 (41). This strong reduction of RPA2 hyperphosphorylation in HU-treated shPARG did not result from a delayed phosphorylation, since lower levels were observed all along the release from an HU treatment of 24 h (Figure 4B). In agreement with previous reports (36,37), the phosphorylation of RPA2 on S4S8 was mainly due to DNA-PK since cell pre-treatment with the specific DNA-PK inhibitor NU7441 at 10 μM strongly decreased RPA2 hyperphosphorylation in both shCTL and shPARG (Figure 4C, compare lane 7 with 5 and lane 8 with 6). The deficiency in RPA2 S4S8 phosphorylation in shPARG cells was not due to a defective DNA-PK activity, since DNA-PK was still capable to phosphorylate its other substrate H2AX after HU in these cells, as illustrated by the strong decrease of γH2AX in shPARG cells treated with NU7441, similar to shCTL cells (Figure 4C, compare lane 8 with lane 6).

To investigate in more details the involvement of PARG in the regulation of RPA2 hyperphosphorylation, we examined by immunofluorescence RPA foci formation in both cell lines. We observed RPA2 and P-S4S8-RPA2 foci in shCTL cells after 24 h of HU treatment, as expected (Figure 4D and E, respectively). In contrast, only shPARG cells with undetectable or low PAR (PAR−/+), but not high PAR (PAR++) showed RPA2 or P-S4S8-RPA2 foci (Figure 4D and E, respectively). A clear inverse correlation was observed between RPA2 or P-S4S8-RPA2 foci formation and PAR accumulation since 75% of the shPARG cells with high PAR levels (PAR++) did not show P-S4S8-RPA2 foci after the HU treatment, and almost all of them after the release (Figure 4F). To determine whether PARG modulates the recruitment of RPA2 onto chromatin, we next evaluated the chromatin loading of RPA2 and its hyperphosphorylated form in response to 2 mM HU for 24 h (Figure 4G). Cell fractionation was performed according to Cheng *et al.* (31) using successive detergent extractions to separate loosely bound proteins (fractions I and II) from less extractable and chromatin-enriched fractions (fractions III and IV, respectively). GAPDH and histone H4 were used as markers of soluble and chromatin fractions, respectively (Supplementary Figure S1). In HU-treated shCTL cells, RPA2 accumulated and persisted in the chromatin-enriched fraction in its hyperphosphorylated form (Figure 4G, lane 4). In contrast, in HU-treated shPARG cells, RPA2 remained mainly in the early extracted fractions in its unphosphorylated form (Figure 4G, lane 8). Taken together, these results reveal that PARG deficiency impairs RPA2 hyperphosphorylation on S4S8 and RPA recruitment onto chromatin in response to a prolonged HU treatment.

### Homologous recombination is impaired in high PAR HU-treated shPARG cells

To evaluate the consequence of PARG deficiency and defective RPA2 loading onto chromatin on the HR-mediated recovery from prolonged replication fork stalling, we examined the recruitment of RAD51 onto chromatin and formation of RAD51 foci following an HU treatment of 24 h and after different release time points. By subcellular fractionation, we observed that the presence of RAD51 in both fractions III and IV increased with time after the release from HU treatment in both shCTL and shPARG cell lines (Figure 5A). RAD51 loading onto chromatin was, however, not obviously altered in shPARG cells compared to shCTL cells (Figure 5A). By immunofluorescence, RAD51 foci were detected in shPARG cells with undetectable or low PAR (PAR−/+) cells at comparable levels than in shCTL cells (Figure 5B and C) confirming that HR-mediated repair of HU-induced DSB is globally not affected in shPARG cells. However, cells with high PAR levels (PAR++) were almost devoid of RAD51 foci, in agreement with the defective RPA2 foci formation, indicating that the HR-mediated repair process is non-functional in this population of HU-treated shPARG cells (Figure 5B and C). These results suggest that HR-mediated repair of HU-induced persistently stalled replication forks is functional when only limited amounts of PAR are produced in HU-treated shPARG. In contrast, shPARG cells accumulating high PAR levels could not recover replication-induced DSB by HR.

To evaluate whether PARG is required for the HR process itself, we used the HR-inducible U2OS-DR-GFP cells stably expressing the mCherry-*I-Sce*I-GR fusion protein (28). Reconstitution of GFP after *I-*SceI-dependent HR was monitored by flow cytometry in cells transfected with siRNA targeting PARG or BRCA1, or scramble siRNA (Figure 5D). The knockdown efficiency was verified by western blot and RT-qPCR (Supplementary Figure S2). BRCA1 depletion dramatically reduced HR efficiency to 0.12 ± 0.01 compared to scrambled control siRNA (SCR), as expected for a key actor of this repair process and according to Ransburg *et al.* (42) and PARG depletion reduced HR to 0.76 ± 0.11 (*P* = 0.008 versus SCR). This result indicates that PARG is not essential but facilitates the HR process.

### Persistence of massive ssDNA in high PAR HU-treated shPARG cells

The next step was to identify the cause of the defective RPA2 loading onto chromatin in shPARG cells upon prolonged replicative stress. Since S4S8-phosphorylation of RPA2 is commonly used as a marker of effective DNA end resection (39,40), a flawed resection activity could explain the reduced RPA2 hyperphosphorylation and RPA loading detected in HU-treated shPARG cells. We thus looked at ssDNA formation using the assay based on BrdU immunodetection in the absence of DNA denaturation (30) (Supplementary Figure S3A). BrdU was not detected in untreated cells, but could be detected after prolonged HU treatment in both shCTL and shPARG cells (Supplementary Figure S3B, middle and right panels, respectively), suggesting the formation of ssDNA. Quantification of all BrdU-positive cells revealed no differences between HU-treated shCTL and shPARG cells at any time point examined after the release (Figure 6A and B), suggesting that resection is globally functional in HU-treated PARG-deficient cells. However, when focusing on high BrdU-positive cells (BrdU++, Figure 6A) that were detected in both cell lines at similar proportion just after the HU treatment, we observed that these cells persisted several hours after the release only in shPARG cells whereas they gradually decreased in number and almost disappeared 8 h after the release from HU treatment in shCTL cells (Figure 6C). These persisting strong BrdU-positive shPARG cells were those displaying high amount of PAR (PAR++) and still present after the release from HU-treatment (Figure 6A). Two non-exclusive hypotheses could explain this strong BrdU signal: an increased resection or a global destabilization of chromatin structure due to the accumulation of PAR favoring DNA accessibility to immunodetection (43).

To challenge the first hypothesis, and since PARP-1 was shown to facilitate the recruitment of the resecting enzyme MRE11 to DSB and at stalled replication forks (12,44), we examined the HU-induced recruitment of MRE11 onto chromatin by subcellular fractionation. Mobilization of MRE11 to less extractable and chromatin fractions was comparable for both cell lines (Figure 6D), supporting the fact that resection is globally not affected by the absence of PARG in conditions of prolonged HU treatment. To evaluate the contribution of MRE11 resecting activity in HU-treated cells, we inhibited MRE11 nuclease activity with 20 μM mirin. Mirin strongly decreased the phosphorylation of RPA2 on S4S8 in HU-treated shCTL cells, as expected (Supplementary Figure S4), but did not significantly decrease the level of S4S8 phosphorylation that was already low in HU-treated shPARG cells (Supplementary Figure S4). In contrast, mirin significantly decreased the HU-induced phosphorylation of H2AX in both shCTL and shPARG cells (Supplementary Figure S4), indicating that MRE11 activity is functional in both HU-treated cell lines and partly contributes to the phosphorylation of H2AX, most likely via its activating effect on ATM (45). By immunofluorescence, MRE11 foci were rather difficult to detect after HU treatment in our cellular model, precluding from quantitative analyses. However, we could clearly observe that in HU-treated shPARG cells, MRE11 foci could be detected in low PAR producing cells as well as in shCTL cells, but not in the high PAR-positive shPARG cells (Figure 6E), suggesting impaired recruitment in these cells, Using mirin, we evaluated the contribution of MRE11 activity on the formation and clearance of HU-induced high-BrdU-positive (BrdU++) cells (Figure 6F). Mirin reduced the proportion of BrdU++ cells in both cell lines but the decrease was statistically significant only in shCTL cells at 24 h of HU. At 2 and 8 h after the release from HU, BrdU++ cells almost disappeared in shCTL cells. In shPARG cells, the presence of mirin reduced, however not significantly, the proportion of BrdU++ cells, never reaching the low level of BrdU++ cells observed in shCTL cells, indicating that MRE11 activity cannot be solely responsible for the high persistent ssDNA in these cells. Taken together, these results indicate that resection is globally not affected in HU-treated shPARG cells with low PAR levels. In contrast, in the high PAR shPARG cells, resection seems however affected (MRE11 foci formation is impaired and only moderate effect of mirin), and can therefore not explain the delayed clearance of cells with massive ssDNA.

An alternative explanation for the delayed clearance of high BrdU cells in HU-treated shPARG cells could originate from the massive PAR level directly increasing the BrdU accessibility. To test this possibility, we scored the formation and clearance of BrdU++ cells when HU treatment was performed in the presence of the PARP inhibitor KU0058948 at 200 nM (+PARPi, Figure 6F). Whereas PARP inhibition had almost no impact on the proportion of BrdU++ cells in both cell lines treated with HU for 24 h, it dramatically reduced the proportion of BrdU++ cells in shPARG cells released for 2 h and 8 h from the HU treatment, now reaching the same level of BrdU++ cells observed in shCTL cells. This result supports the hypothesis that massive PAR level directly increases ssDNA accessibility.

### High PAR prevents the accumulation of RPA2 onto chromatin in HU-treated shPARG cells

**Figure 7.**
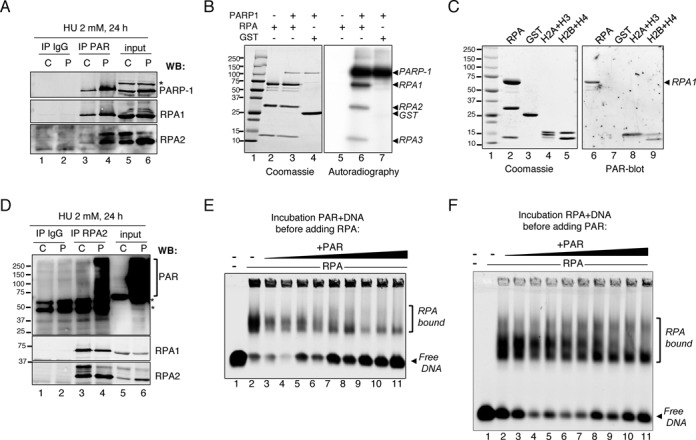
RPA binding to DNA is directly affected by PAR. (**A**) Anti-PAR antibodies co-immunoprecipitated RPA2 and RPA1 subunits of RPA at higher levels from HU-treated shPARG cells compared to shCTL cells. Cell extracts from shCTL (C) and shPARG (P) cells treated with 2 mM HU for 24 h were immunoprecipitated with anti-PAR antibodies. The presence of PARP-1, RPA1 and RPA2 in the anti-PAR immunoprecipitates (IP PAR, lanes 3 and 4), in the control immunoprecipitates using rabbit anti-mouse antibody (IP IgG, lanes 1 and 2) and in the input (lanes 5 and 6, corresponding to 5% of the lysate) was assessed by western blotting using the indicated antibodies. The asterisk points to a non-specific band. (**B**) PARP-1 PARylated by RPA mostly on RPA1 subunit. The trimeric RPA (2.5 μg, lanes 2, 3, 5 and 6) was incubated with 0 (lanes 2 and 5) or 500 ng of PARP-1 (lanes 3 and 6) for 30 min at 25°C in the presence of ^32^P-NAD^+^ and DNAse I-activated calf thymus DNA. As negative control, PARP-1 was incubated with GST (2 μg) (lanes 4 and 7). Reaction products were analysed by 8–20% SDS-PAGE and autoradiography (right panel) of the Coomassie blue-stained and dried gel (left panel). Lane 1: molecular weight marker. (**C**) RPA binds non-covalently to PAR. RPA complex (2.5 μg, lanes 2 and 6), GST (2 μg, lanes 3 and 7), H2A and H3 (1 μg each, lanes 4 and 8) and H2B, H4 (1 μg each, lane 5 and 9) were separated on 8–20% SDS–PAGE and either stained with Coomassie blue (left panel) or blotted onto nitrocellulose membrane, renaturated and incubated with free PAR. Bound PAR was detected by immunodetection using anti-PAR antibody (right panel, PAR-blot). Lane 1: molecular weight marker. (**D**) PARylated proteins are co-immunoprecipitated with RPA2 in HU-treated shPARG cells. Cell extracts from shCTL (C) and shPARG (P) cells treated with 2 mM HU for 24 h were immunoprecipitated with anti-RPA2 antibodies as described in “*Material and Methods*” section. The presence of PARylated proteins, RPA1 and RPA2 in the anti-RPA2 immunoprecipitates (IP RPA2, lanes 3 and 4), the control immunoprecipitates using mouse anti-HA antibody (IP IgG, lanes 1 and 2) and the input (lanes 5 and 6, corresponding to 5% of the lysates) was assessed by western blotting using the indicated antibodies. The asterisk points to non-specific bands. (**E and F**) EMSA analyses showing that RPA binding to ssDNA is favored by low levels but counteracted by high levels of PAR. (**E**) PAR competes with DNA for RPA. RPA (1.7 nM) was incubated with 1 nM of a Cy5.5-oligodT probe (50-mer) and increasing concentrations of PAR (lanes 3–11: from 8 ng in lane 3, doubled each point to 256 ng in lane 12) for 30 min at RT, complexes were separated by 1% native agarose gel electrophoresis and observed at 680 nm on Odyssey Infrared Imaging System. Lane 1: DNA only; lane 2: no PAR. **F**. High PAR levels dislodge RPA from DNA. RPA (7 nM) was pre-incubated with 2.5 nM of a Cy5.5-oligodT probe for 15 min before the addition of increasing concentrations of PAR (from lane 3 to 11: 4, 8, 16, 32, 48, 64, 96, 128, 192 ng, respectively) and incubation for 30 min at 23°C. Complexes were separated by 1% native agarose gel electrophoresis and observed at 680 nm on Odyssey Infrared Imaging System. Lane 1: DNA only; lane 2: no PAR.

If ssDNA accessibility is increased in HU-treated shPARG cells with high PAR level, then why RPA loading onto this ssDNA is impaired? A likely hypothesis is that high PAR/PARP-1 activity could directly act on RPA. Both RPA subunits RPA1 and RPA2 have been shown to be targets for PARylation, particularly in response to DNA damage (46,47). To test this hypothesis, we performed an immunoprecipitation of HU-treated shCTL and shPARG cell extracts using an anti-PAR antibody and analysed the co-immunoprecipitation of PARP-1 and RPA subunits. PARP-1 was precipitated at higher levels in HU-treated shPARG cells than in HU-treated shCTL cells, as expected (Figure 7A). Both RPA1 and RPA2 were pulled down more efficiently from HU-treated shPARG cells than from HU-treated shCTL cells, suggesting that RPA could be highly PARylated in HU-treated shPARG cells. Alternatively, the non-covalent binding of RPA to PAR would also lead to increased co-immunoprecipitation with anti-PAR antibody in HU-treated shPARG cells. However, the smear formed by RPA1 immunoprecipitated with anti-PAR antibodies strongly supports RPA1 PARylation. We then evaluated whether RPA could be PARylated or could bind non-covalently to PAR *in vitro*, using recombinant proteins. Incubation of recombinant trimeric RPA (48) with PARP-1 in the presence of ^32^P-NAD^+^ and fragmented DNA revealed that all three RPA subunits, but not the negative control GST, could be efficiently PARylated by PARP-1, with RPA1 being the major target for PARylation (Figure 7B). PAR-blot assays revealed that only RPA1 was able to bind PAR non-covalently akin to the positive controls histones H2B, H3 and H4 (Figure 7C). Altogether, these results indicate that RPA is not only a target for PARylation, it can also bind PAR non-covalently. Next, we immunoprecipitated RPA2 from HU-treated shCTL and shPARG (Figure 7D) and revealed that although RPA1 could be co-immunoprecipitated in both cells lines, PARylated proteins detected with the anti-PAR antibody as a smear were co-immunoprecipitated at higher levels in shPARG cells compared to shCTL cells (Figure 7D). This demonstrates that RPA associates with PARylated proteins in HU-treated shPARG. Finally, we evaluated the impact of PAR on RPA binding to ssDNA using electrophoretic mobility shift assays (EMSA) with recombinant trimeric RPA and a single-stranded 50-mer oligo-dT. Whereas the presence of low amounts of free PAR molecules appeared to favor RPA binding to DNA (Figure 7E, lanes 3 and 4, free DNA is decreased), higher amounts of PAR had the opposite effect decreasing RPA binding, indicated by the increase in free DNA (lanes 5–11). When RPA was first pre-incubated with DNA before the addition of PAR, results were comparable, with low amounts of PAR rather stimulating RPA binding to DNA (Figure 7F, lanes 3–7, note the significant decrease in free DNA) whereas higher PAR levels decreased RPA binding to DNA (lanes 8–11, free DNA is increased). Taken together, these *in vitro* and *in vivo* experiments suggest that low PAR level does not affect RPA accumulation onto ssDNA and could even favor it, whereas strong PARylation prevents RPA accumulation onto ssDNA, because of both increased PARylation of RPA and non-covalent binding of RPA to the excessive PAR.

### PARP inhibition restores RPA2 hyperphosphorylation and mobilization onto chromatin in HU-treated shPARG cells

**Figure 8.**
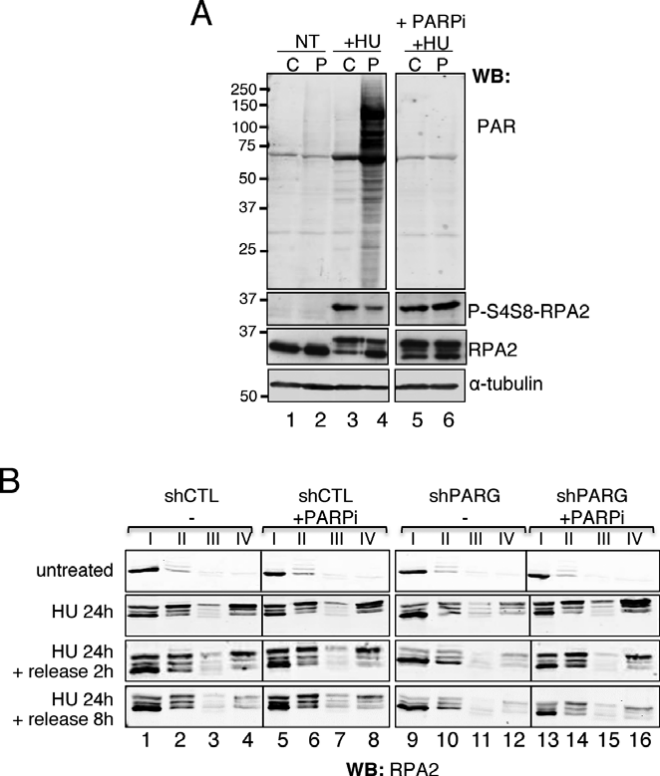
PARP inhibition restores the hyperphosphorylation and chromatin association of RPA2 in HU-treated shPARG cells. (**A**) Restoration of the hyperphosphorylation of RPA2 at S4S8 in shPARG cells treated with 2 mM HU for 24 h in the presence of a PARP inhibitor. Equivalent amount of cell extracts prepared from shCTL (C) or shPARG (P) cells untreated (NT) or treated with 2 mM HU for 24 h (+HU) in absence or presence of 200 nM of the PARP inhibitor KU0058948 (+PARPi) as described in *“Material and Methods”* section, were analysed by western blotting using the indicated antibodies. (**B**) RPA2 loading onto chromatin is restored in shPARG cells treated with 2 mM HU for 24 h in the presence of a PARP inhibitor. shCTL and shPARG cells either untreated or treated with 2 mM HU 24 h and released into fresh medium for 2 or 8 h, in the absence (−) or presence of 200 nM of the PARP inhibitor KU0058948 (+PARPi) were collected, fractionated as described in the “*Materials and Methods*" section leading to fractions I to IV. Equivalent cell numbers of each fraction were analysed by western blotting using an anti-RPA2 antibody.

To further examine the contribution of PAR in the decreased recruitment of RPA onto chromatin, we treated shCTL and shPARG cells with 2 mM HU for 24 h in the presence of the PARP inhibitor KU0058948 at 200 nM. The complete prevention of PAR synthesis in HU-treated shPARG cells confirmed efficient inhibition of PARPs (Figure 8A, lanes 5 and 6). PARP inhibition restored RPA2 hyperphosphorylation on S4S8 (Figure 8A, compare lanes 6 with lane 4) and its accumulation on the chromatin fraction (Figure 8B, compare line 16 with line 12) in HU-treated shPARG cells. Altogether, these results demonstrate that the accumulation of PAR in shPARG cells upon prolonged replicative stress dramatically affects RPA2 loading onto chromatin and hyperphosphorylation.

## DISCUSSION

In this work we have shown that PARG is dispensable for DNA replication in unstressed conditions and from recovery from transiently stalled replication forks. Indeed, PARG-deficient cells displayed normal S-phase progression after release from a short HU treatment and normal checkpoint activation (phosphorylation of Chk1 at S345 and RPA at S33). The similar phosphorylation of RPA2 at S33 in shPARG and shCTL cells implies that RPA is normally recruited to the stalled replication forks, reflecting unaltered stabilization of these stalled forks and resection. This is in agreement with the observed similar ssDNA formation monitored by BrdU staining in both cell lines after 4 h of HU treatment. Only limited PAR is produced in shPARG cells after short HU incubation. Recently, PARP-1 activity was reported to promote the accumulation and stabilization of regressed replication forks upon exposure to sub-lethal doses of topoisomerase I inhibitors, to prevent fork collapse and DSB formation but also premature restart in conditions of mild replication stress (20). In addition, PARP-1 was shown to protect HU-mediated stalled replication forks from excessive resection by MRE11 (17). Even if nucleotide deprivation by HU and topoisomerase I/DNA covalently linked-lesions are not equivalent, the fact that PARG deficiency has no impact on recovery from HU-mediated transient replicative stress suggests that a moderate increase in PAR production by PARP-1 would not be detrimental to cellular processes. This is in agreement with our previous findings showing that PAR produced in shPARG cells in the absence of massive exogenous genotoxic stress had rather a protective effect toward genome integrity and telomere stability (6).

However, PARG deficiency delays recovery from persistent replication stress triggered by prolonged HU treatment, with some cells even not resuming S-phase progression. These blocked cells displayed high PAR levels and continuous phosphorylation of Chk1 on S345, supporting the fact that they did not resume cell cycle progression. Our results are in agreement with the recent findings that replicative stress-mediated Chk1 activation can be directly regulated by PAR molecules (13). These HU-treated shPARG cells with high PAR levels also showed high γH2AX, suggesting increased replication fork collapsing upon persistent replicative stress. A recent study from Shirai *et al.* (22) also reported an S-phase arrest and an increase in DSB formation in Parg^−/−^ mouse ES cells or PARG knockdown pancreatic cancer cells treated with an alkylating agent which leads to high PAR levels in these PARG-deficient cells. Similarly, we observed that when shPARG cells were treated in S-phase with the alkylating agent *N*-methyl *N*′-nitro-*N*-nitrosoguanidine (MNNG), recovery of S-phase progression was altered and some cells were not able to restart cell cycle progression at all (data not shown), akin to what was observed upon prolonged HU treatment. Therefore, accumulation of high PAR levels upon PARG deficiency can reflect the accumulation of DSB in S-phase, arising from conversion of SSB to DSB upon alkylation treatment or from the collapse of persistently stalled forks upon prolonged HU treatment.

Prolonged HU treatment leading to replication fork collapse triggers the hyperphosphorylation of RPA2 on S4S8 (33–35,37,38,49). P-S4S8 RPA2 hyperphosphorylation was indeed observed in shCTL cells but strongly impaired in shPARG cells. This defect did not originate from a defective activity of the main kinase involved in this phosphorylation, DNA-PK (37,50,51), since the HU-induced phosphorylation of γH2AX in shPARG cells was as sensitive to the DNA-PK inhibitor NU7441 as that in shCTL cells. This observation also confirms that DNA-PK is one of the main trigger of H2AX phosphorylation at persistently stalled forks, the other kinase being ATM (36). The fact that increased PARylation in shPARG cells does not alter DNA-PK activity is in agreement with a previous study reporting that DNA-PK is rather activated by PARylation (52,53). RPA2 associated with ssDNA is more accessible to kinases activity than free protein, a consequence of the structural changes of its N-terminal extremity (54,55). Therefore, the lack of HU-induced RPA2 hyperphosphorylation in shPARG suggests that loading of RPA onto ssDNA is defective in these cells. Indeed, RPA2 accumulation on chromatin was dramatically reduced in shPARG cells at conditions of persistent replication fork stalling. The investigation of RPA2 and P-S4S8-RPA2 foci formation by immunofluorescence revealed that shPARG cells with high PAR levels were the most affected in RPA2 mobilization. A defective resection was the first possible hypothesis we tested. Looking at the global cell population of HU-treated shPARG, MRE11 accumulation on chromatin appeared to be not affected compared to shCTL cells. This is consistent with PARP-1/PAR-facilitated recruitment of MRE11 to collapsed replication forks as described previously (12,44), MRE11 possessing a canonical functional PAR-binding motif (44,56). In agreement, BrdU immunodetection showed similar amounts of ssDNA formed in both cell lines, suggesting that DNA resection is globally not affected in shPARG cells. Interestingly, the HU-treated shPARG having high PAR levels showed impaired formation of MRE11 foci and RPA2 foci but in contrast, a strong and persistent BrdU staining, indicating high ssDNA amount in these cells. The inhibition of MRE11 nuclease activity by mirin reduced the proportion of cells with high BrdU in both cell lines but the decrease was statistically significant only in the shCTL cells. Importantly, whereas the highly BrdU-positive cells rapidly disappeared in shCTL after the release from the HU treatment, they persisted in shPARG, in the absence or presence of mirin, never reaching the low levels of shCTL cells. This suggests that, even if MRE11 could contribute to the formation of this high BrdU signal observed in shPARG cells during the HU treatment, it cannot solely explain the persistence of high ssDNA level in high PARG shPARG cells released from the HU treatment.

Even if we cannot exclude that other resecting enzymes (ExoI, DNA2) could eventually compensate the lack of recruitment of MRE11 to collapsed forks in the high PAR shPARG cells, the persistent high BrdU signal in these cells could rather reflect the accumulation of accessible ssDNA due to high PAR content. An increased DNA accessibility due to massive PAR level has been already reported previously in Parg^−/−^ trophoblast stem cells (43). This hypothesis is supported by the complete clearance of these BrdU++ cells in shPARG cells treated with HU and released in the presence of a PARP inhibitor. Even if PARP inhibition could have a direct inhibitory effect on the MRE11-dependent resection, as reported previously (12), the fact that the PARP inhibitor is more efficient than mirin to reduce the proportion of BrdU++ shPARG cells during the release suggests a broader effect of PAR, which is the direct effect on RPA. Nevertheless, this result implies that in the context of high PAR cellular content, ssDNA monitoring by BrdU immunodetection should be considered with caution as a marker of ssDNA generated by resection at DSB. Our study shows that the high levels of PAR could directly impact on RPA loading onto ssDNA. All three RPA subunits could be efficiently PARylated by PARP-1 *in vitro*, in agreement with Eki and Hurwitz (46) and RPA was co-immunoprecipitated with anti-PAR antibodies at higher levels from shPARG cells compared to shCTL after HU treatment, suggesting that the RPA complex was highly PARylated in shPARG cells. This is also in agreement with previous reports showing that RPA1 and RPA2 are PARylated in response to genotoxic stress (47) and that RPA1 is pulled down with anti-PAR antibody (57). In addition, PAR was able to non-covalently bind RPA and could even stimulate RPA binding to ssDNA *in vitro* when present at low levels. This is in agreement with the study of Bryant *et al.* (12) that showed that PARP-1 absence or inhibition decreased three times but did not abolish the formation of RPA2 foci in HU-treated U2OS cells or Parp1^−/−^ cells, suggesting that PARP-1/PAR could favor the formation of some, but not all, RPA2 foci. Our study now shows in addition that when PAR is present at high levels, it can even have the opposite effect, impacting RPA2 foci formation *in vivo* and RPA association with ssDNA *in vitro*. Our *in vitro* data fully support our *in vivo* observations and suggest that in HU-treated shPARG cells with high PAR levels, PAR molecules could directly counteract RPA binding to ssDNA.

Supporting this hypothesis, RPA2 recruitment onto chromatin was restored in shPARG cells when the prolonged HU-treatment was performed in the presence of a PARP inhibitor, similar to what was observed in HU-treated shCTL cells, and RPA2 hyperphosphorylation on S4S8 was also completely recovered. We observed an analogous decrease in RPA2 hyperphosphorylation in MDA-MB231 breast cancer cells transiently depleted in PARG by siRNA and treated with 2 mM HU for 24 h, and this hyperphosphorylation was restored in the presence of a PARP inhibitor (data not shown). Therefore, our study indicates that deregulated PAR accumulation directly impairs RPA2 loading onto chromatin and hyperphosphorylation at collapsed replication forks. A recent elegant study using models of RPA exhaustion demonstrated that accumulation of RPA on chromatin occurs before DSB generation at stalled forks, and that these DSB are formed in ssDNA regions not sufficiently protected by RPA (58,59). A correlation between RPA exhaustion and increased DSB formation was clearly established. It is therefore very likely that the shPARG cells with high PAR levels mimic a situation of RPA exhaustion: the PAR produced at stalled forks progressively accumulates in PARG-deprived cells and thus impacts RPA loading onto chromatin. Thus, the more RPA is prevented from loading, the more uncovered ssDNA stretches are produced, the more DSB are formed, and the more PAR is produced. Such an amplification loop would explain this population of HU-treated shPARG cells displaying excessive levels of PAR and γH2AX but no RPA2 and RAD51 foci.

RPA2 and MRE11 are required for RAD51 recruitment at persistently stalled forks to trigger HR-mediated repair of the formed DSB (36,49,60). In light with this, the lack of MRE11, RPA2 and RAD51 foci formation in the HU-treated high PAR shPARG cells suggests that the homology-directed repair of the collapsed forks is abolished in these cells. PARP-1 depletion or inhibition was reported to have no effect on the I-*Sce*I-induced HR process (11,16,61) but to increase the nick-induced HR (61), a likely explanation for the increased SCE occurring spontaneously or upon treatment with nick-inducing genotoxic agents. In contrast, Parg^−/−^ mouse ES cells were not more prone to spontaneous or MMS-induced SCE than Parg^+/+^ cells (62) suggesting no increase in nick-induced HR, whereas our results show that PARG depletion reduces the HR-mediated repair efficiency of I-*Sce*I-induced DSB. Even if the HR-processing of DSB induced by collapsed forks or by endonucleases is not strictly comparable, PAR accumulation in a context of PARG deficiency is detrimental in both situations, supporting the long-lasting hypothesis of anti-recombination properties of PAR.

In summary, our study reveals that PARG is dispensable for cell recovery from transient replicative stress but necessary to regulate PAR levels to avoid massive PAR production and accumulation upon prolonged replicative stress that leads to replication fork collapse and DSB. Extensive PAR prevents RPA accumulation onto chromatin at replication foci, affecting its interaction with ssDNA. The increased sensitivity of PARG-deficient cells to the anti-neoplastic agent HU that blocks replication supports the concept of PARG inhibition to potentialize cancer therapies relying on genotoxic drugs as previously proposed (6,14,63,64).

## SUPPLEMENTARY DATA

Supplementary Data are available at NAR Online.

SUPPLEMENTARY DATA
