# Supplementary material for: PARG is dispensable for recovery from transient replicative stress but required to prevent detrimental accumulation of poly(ADP-ribose) upon prolonged replicative stress
Source: Nucleic Acids Res. 2014 Jun 7;42(12):7776–92. doi: 10.1093/nar/gku505 (PMC4081103; doi:10.1093/nar/gku505)
Supplement: SUPPLEMENTARY DATA [file supp_42_12_7776__index.html]

PARG is dispensable for recovery from transient replicative stress but required to prevent detrimental accumulation of poly(ADP-ribose) upon prolonged replicative stress — PARG is dispensable for recovery from transient replicative stress but required to prevent detrimental accumulation of poly(ADP-ribose) upon prolonged replicative stress — SUPPLEMENTARY DATA 

# PARG is dispensable for recovery from transient replicative stress but required to prevent detrimental accumulation of poly(ADP-ribose) upon prolonged replicative stress

## SUPPLEMENTARY DATA

**Files in this Data Supplement:**

- SUPPLEMENTARY DATA
