# Supplementary material for: PARG is dispensable for recovery from transient replicative stress but required to prevent detrimental accumulation of poly(ADP-ribose) upon prolonged replicative stress
Source: Nucleic Acids Res. 2014 Jun 7;42(12):7776–92. doi: 10.1093/nar/gku505 (PMC4081103; doi:10.1093/nar/gku505)
Supplement: SUPPLEMENTARY DATA [file supp_gku505_nar-00491-d-2014-File010.pdf]

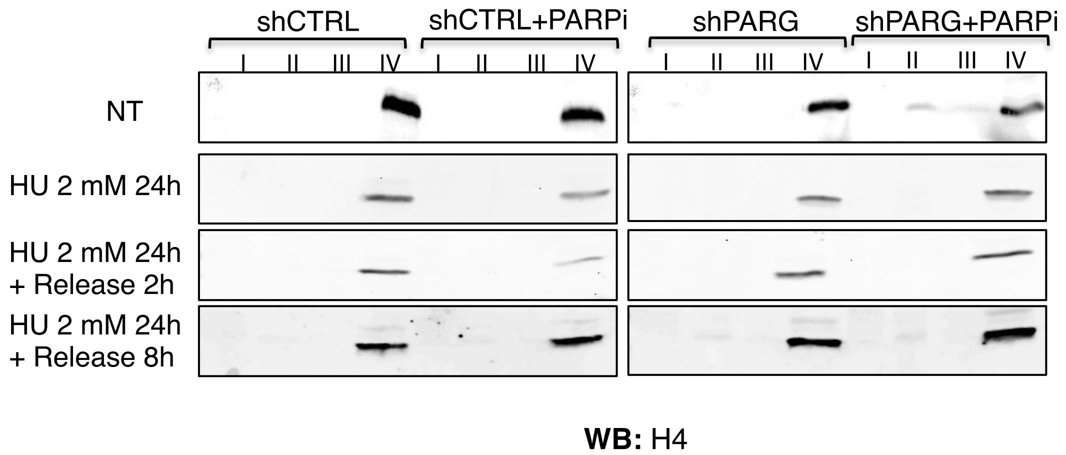

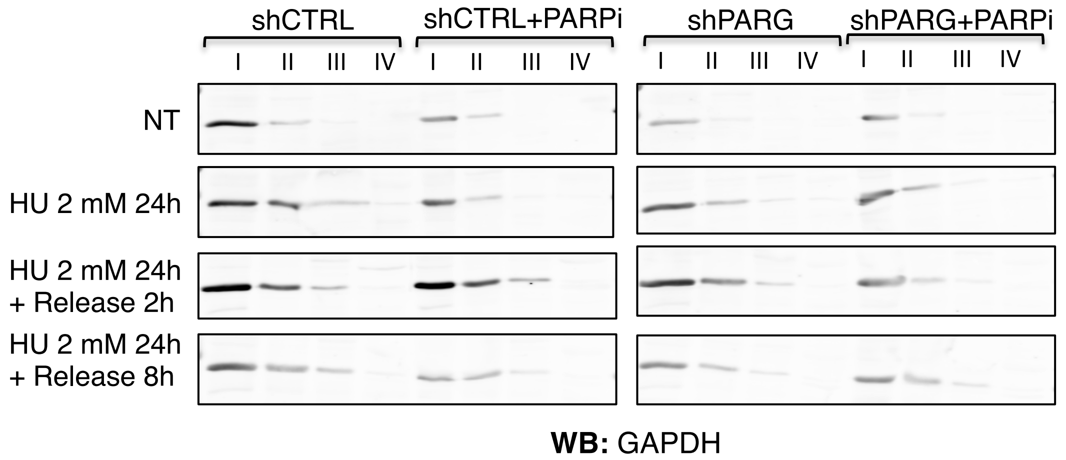

Supplementary Figure 1. Cell Biochemical fractionation. Same number of shCTL and shPARG cells untreated (NT) or treated with 2 mM HU for 24 hours and further released into fresh medium for 2 or 8 h, was collected, fractionated as described in the "Materials and Methods" section leading to fractions I to IV. Equivalent cell numbers of each fraction separated on 4-20% SDS-PAGE and analysed by western blotting using antibodies against cytoplasmic (GAPDH) or chromatin (histone H4) markers.

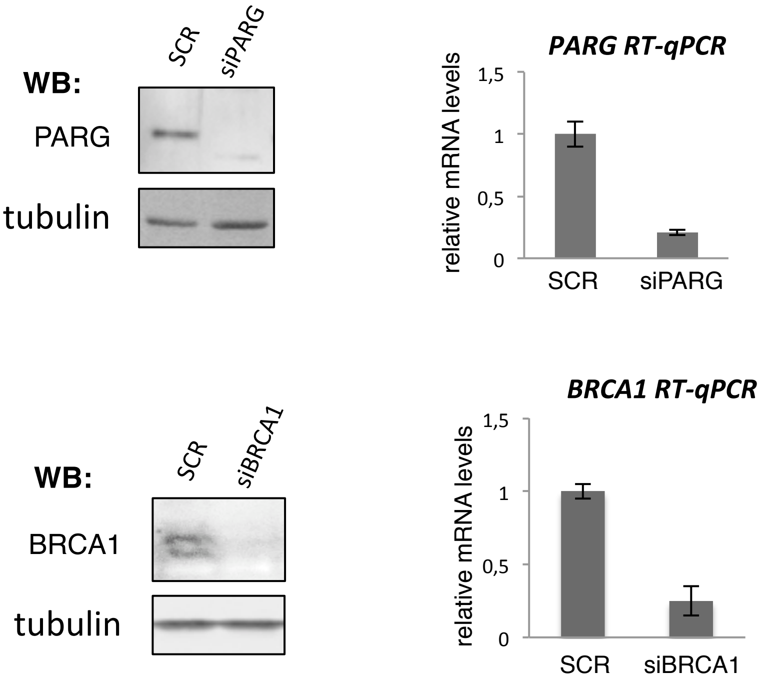

Supplementary Figure 2
U2OS-DR-GFP-mCherry-*I-Sce*I-GR cells were transfected with the indicated siRNA. Left panels: total cell extracts were analysed by western blotting using the indicated antibodies; right panels: mRNA levels of PARG or BRCA1 expression were analysed by RT-qPCR and normalised against GAPDH expression.

Supplementary Material and Methods:

Quantitative RT-PCR
Total RNA was extracted from siRNA-transfected U2OS cells using the RNAeasy kit (Qiagen) according to the manufacturer's protocol. DNase-treated RNA was processed for reverse transcription using the Maxima Reverse Transcriptase (Thermo Scientific) according to the manufacturer's instructions. Real time PCR was performed using the QuantiTect SYBR Green PCR kit following the manufacturer's instructions (Qiagen) combined with the Applied Biosystems StepOne (Life technologies) detection system. The PCR products were analysed with the StepOne Software. The quantity of PCR products was estimated by the relative standard curve method. All samples were analyzed in triplicates and normalized using the *Gapdh* housekeeping gene. The following primer sequences were used :
PARG: Fwd: GAACAAATTTGTACCCAGTGGA, Rev: TCAAAGCTGTAAAGTCCCATTTCTTA
BRCA1: Fwd: BRCA1: Fwd: ACAAGCGTCTCTGAAGACTG, Rev: AGTTCAGCCATTTCCTGCTG
GAPDH: Fwd, ATGTTCGTCATGGGTGTGAA, Rev: GTCTTCTGGGTGGCAGTGAT

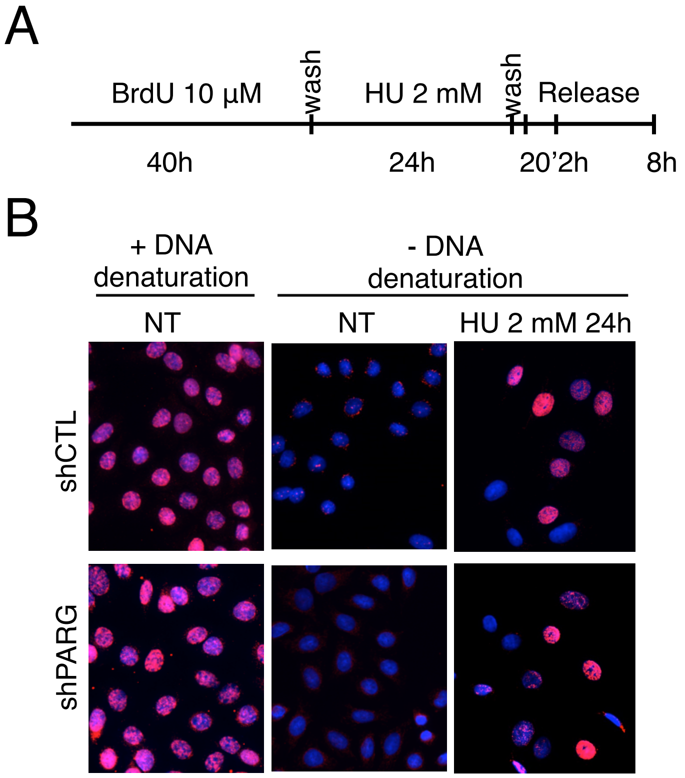

Supplementary Figure 3. BrdU-stained ssDNA immunodetected in shCTL and shPARG cells treated or untreated (NT) with 2 mM HU for 24 h and released into fresh medium for the indicated time points. **A.** Schematic representation of the experiment. BrdU (10 *µ*M) was incorporated for two complete cell cycles before the HU treatment and detected by immunofluorescence using an anti-BrdU antibody as described in *"Material and Methods"* section. **B**. Immunodetection of ssDNA with anti-BrdU antibody. Left panels, representative images of BrdU signal after the DNA denaturation step to demonstrate the incorporation of BrdU in all shCTL and shPARG cells. This step was omitted as indicated in middle and left panels to immunodetect only the ssDNA. DNA was stained with Dapi.

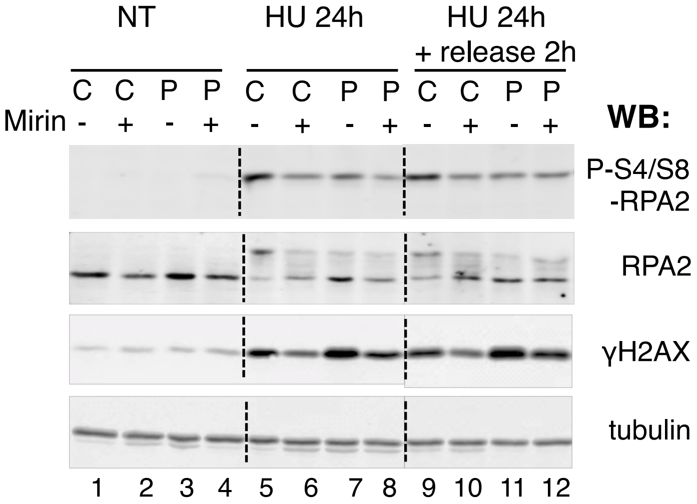

Supplementary Figure 4. Inhibition of MRE11 nuclease activity by mirin decreased the HU-induced phosphorylation of RPA2 on S4S8 in shCTL (C) cells but has little if any effect on the already low phosphorylation level in shPARG (P) cells. Equivalent amounts of total cell extracts prepared after cell treatment with 2 mM HU for 24 h or after release for 2 h were analysed by western blotting using the indicated antibodies. NT: untreated. .
